# Rate of Deceased Kidney Donation From Potential In-Hospital Deaths in the US, 2003-2021

**DOI:** 10.1001/jamanetworkopen.2024.1865

**Published:** 2024-03-11

**Authors:** Jennifer Bragg-Gresham, Ana Laura Licon, Jenna Kiryakos, Rajiv Saran, John P. Roberts

**Affiliations:** 1 Division of Nephrology, Department of Internal Medicine, University of Michigan, Ann Arbor; 2 Department of Epidemiology, School of Public Health, University of Michigan, Ann Arbor; 3 Division of Transplant Surgery, Department of Surgery, University of California San Francisco

## Abstract

This cross-sectional study calculates deceased kidney donation rates in the US using deaths compatible with donation as the metric’s denominator.

## Introduction

While the number of solid organ transplants continues to increase in the US, the shortage of available organs persists. As of 2020, 138 761 patients with end-stage kidney disease were wait-listed for a kidney transplant. However, fewer than 20 000 of these individuals received a deceased kidney transplant.^1^ Although 90% of Americans support organ donation, fewer than 10 donors per 1000 deaths were reported in 2020, with this rate unchanging over time.^2,3^ This trend may be due to using all deaths or traumatic deaths in the metric’s denominator. We investigated this trend using the recent Centers for Medicare & Medicaid Services (CMS) final rule redefinition of deaths compatible with donation, which we hypothesize is a more appropriate denominator and may show different trends.^4^

## Methods

This cross-sectional study combines data from the Scientific Registry of Transplant Recipients (SRTR) and the Centers for Disease Control and Prevention’s (CDC’s) Multiple Cause of Death database. The University of Michigan institutional review board determined this project to be not regulated and exempt from review and informed consent. The study follows the STROBE reporting guideline. We estimated donation rates using the CMS’s final rule to define potential donors between January 1, 2003, and December 31, 2021.^4,5^ This rule defines potential donors as younger than 76 years who died in an inpatient setting with a cause of death compatible with donation. The kidney donation rate among these potential donors was calculated overall and by age, sex, race and ethnicity, and state and reported as the number of donors per 1000 potential donors. Demographic data were from death certificates, as reported in the CDC Multiple Cause of Death and SRTR files, and provided by transplant professionals. Statistical analysis was performed between March 16 and October 1, 2023, using SAS, version 9.4 (SAS Institute Inc). The eMethods in Supplement 1 provide more details on calculations.

## Results

**Figure 1.**
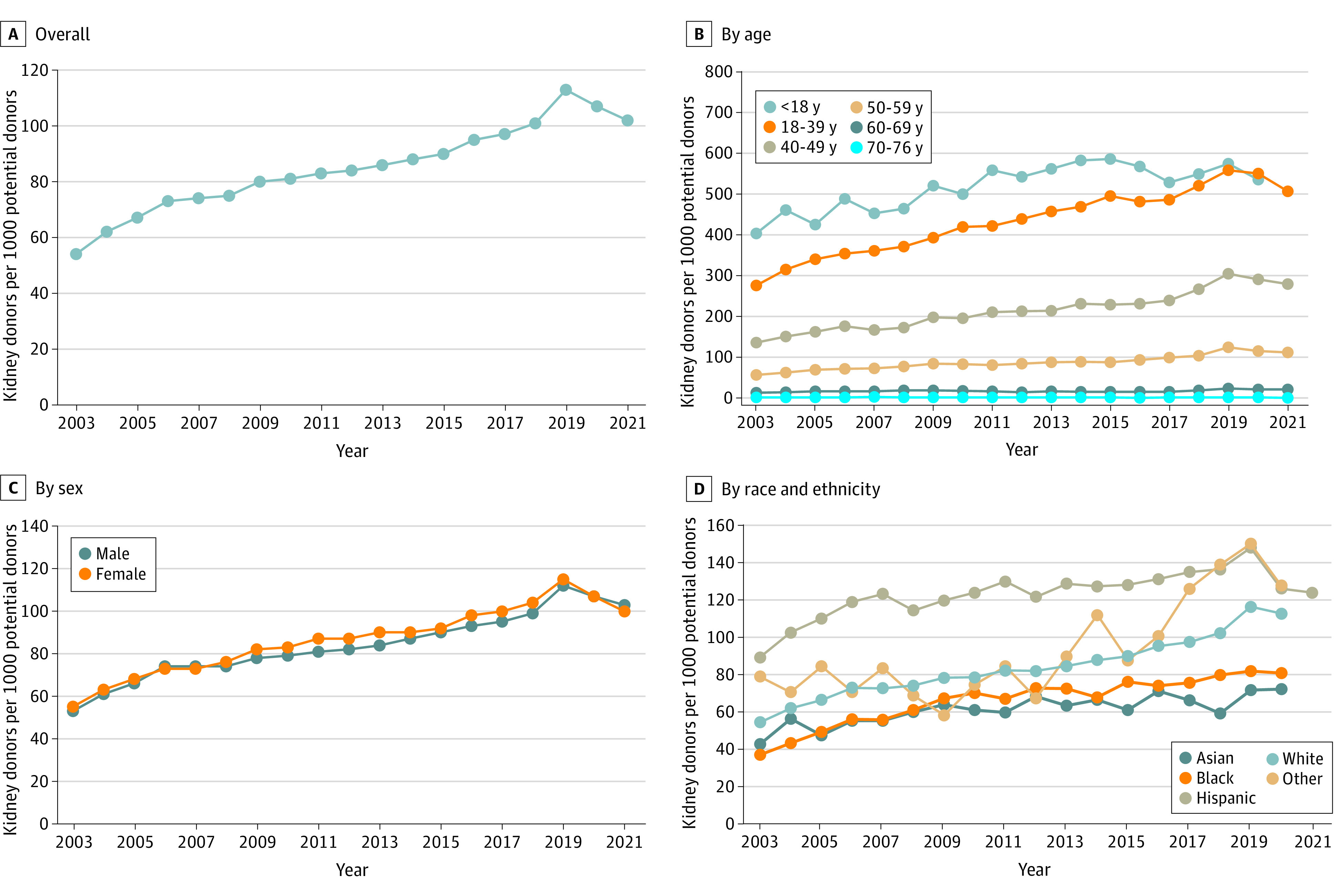
Time Trends in Deceased Kidney Donation Rates, 2003-2021 Other race and ethnicity includes American Indian or Alaska Native, Pacific Islander, multiracial, and unknown.

**Figure 2.**
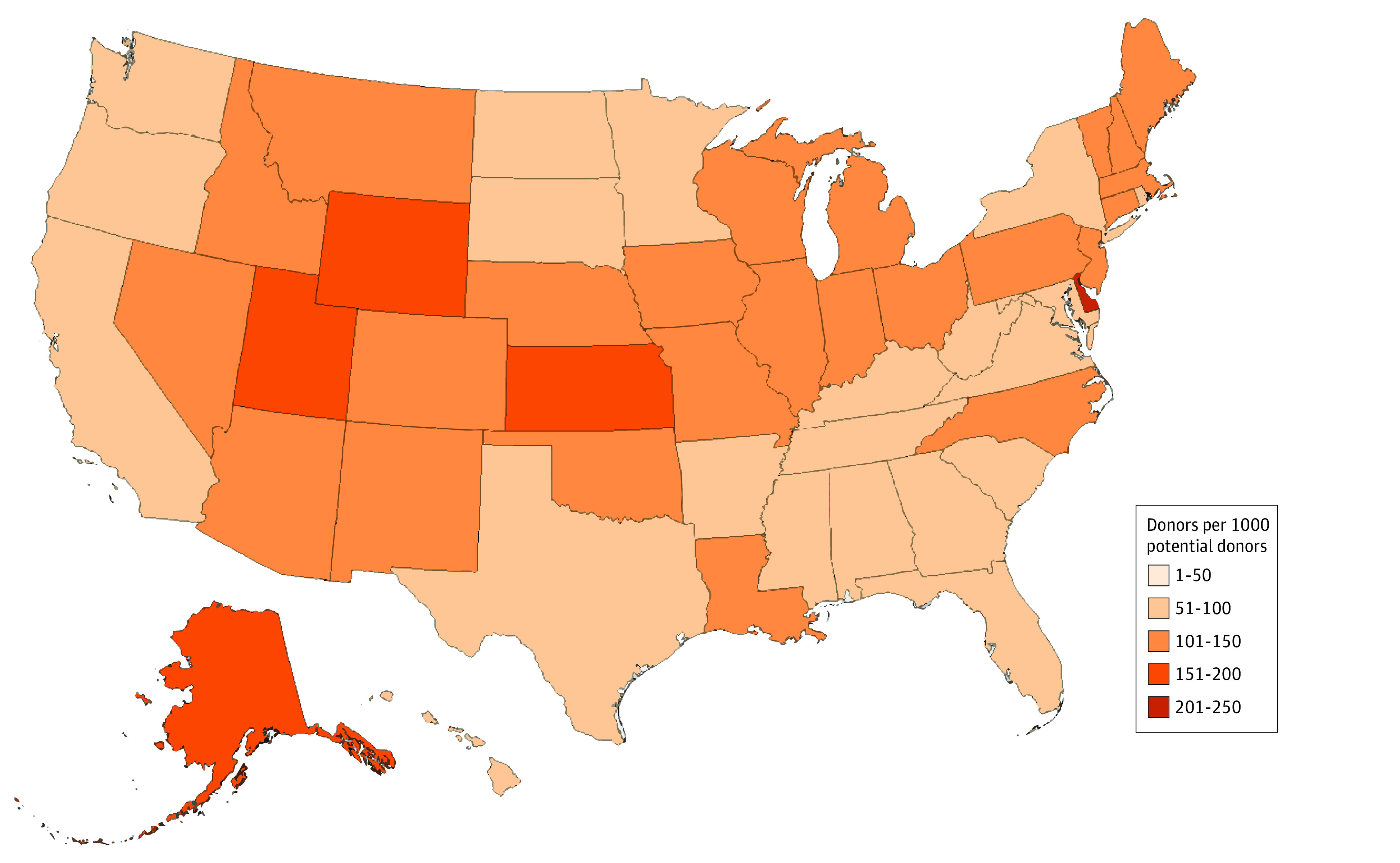
State-Level Deceased Kidney Donation Rate, 2021 Florida multiple-cause-of-death data are only available upon special request and therefore are suppressed in this work.

The number of potential donors increased from 160 119 in 2003 to 183 211 in 2021 and kidney donations from 8654 to 18 691. Overall, kidney donation rates increased from 54 to 102 per 1000 potential donors, with a slight decline during the COVID-19 pandemic, peaking in 2019 at 113 per 1000 (Figure 1). Large differences were seen by age, with the highest rates among younger ages (<18 to 39 years, 500 per 1000 potential donors). No marked difference was observed by sex, although differences were seen by race and ethnicity. Deceased donation rates were highest among Hispanic donors, peaking at 150 per 1000 potential donors, and lowest for Asian and Black donors, peaking at 72 and 81 per 1000 potential donors, respectively. Large geographic differences were observed (Figure 2), with the highest rates in Delaware (>200 per 1000) and lowest in the Southeast and Pacific Coast (≤50 per 1000).

## Discussion

The proportions of kidney donations from potential donors have risen between 2003 and 2021, although they declined during the 2020 to 2021 pandemic period possibly due to suspension of routine health care activities. The highest donation rates were among individuals younger than 39 years and the Hispanic population; the lowest rates were among Asian and Black populations. Our finding of an overall increase in donation rates differs from other published metrics, which used a less specific denominator population and may have been influenced by the opioid epidemic, which continued throughout the study period.^6^ Use of death certificate information for cause of death was a limitation, although no better source is available. These rates should not be influenced by changes in kidney discard rates, as they include only kidneys that were transplanted.

## Conclusions

While rising trends in deceased kidney donations are encouraging, they are barely more than 1 in 10. The kidney transplant community should partner with patient advocates, community-based organizations, policy makers, and other sectors to increase public education and awareness about the importance of kidney donation. Future work should assess interactions among demographics, region, and kidney donations to focus efforts where most needed.

## Notes

Supplement 1. **eMethods.** Details on Calculation of Deceased Donation Rates

Supplement 2.
Data Sharing Statement
