# Error in Figure 1

**DOI:** 10.1001/jamanetworkopen.2024.17050

**Published:** 2024-05-16

In the Research Letter titled “Rate of Deceased Kidney Donation From Potential In-Hospital Deaths in the US, 2003-2021,”^1^ published March 11, 2024, in the key for Figure 1B, the colors for the categories <18 y and 60-69 y were incorrectly swapped. This article has been corrected.^1^
