# Supplementary material for: Rate of Deceased Kidney Donation From Potential In-Hospital Deaths in the US, 2003-2021
Source: JAMA Netw Open. 2024 Mar 11;7(3):e241865. doi: 10.1001/jamanetworkopen.2024.1865 (PMC10928501; doi:10.1001/jamanetworkopen.2024.1865)
Supplement: Supplement 1. — eMethods. Details on Calculation of Deceased Donation Rates [file jamanetwopen-e241865-s001.pdf]

Supplemental Online Content

Bragg-Gresham J, Licon AL, Kiryakos J, Saran R, Roberts JP. Rate of deceased kidney donation from potential in-hospital deaths in the US, 2003-2021. *JAMA Netw Open*. 2024;7(3):e241865. doi:10.1001/jamanetworkopen.2024.1865

eMethods. Details on Calculation of Deceased Donation Rates

This supplemental material has been provided by the authors to give readers additional information about their work.

© 2024 Bragg-Gresham J et al. *JAMA Network Open.*

eMethods. Details on Calculation of Deceased Donation Rates

Denominator: The denominator for deceased donation rates was calculated based on the Centers for Medicare and Medicaid Final Rule, which had the goal to "Revise the Organ Procurement Organization (OPO) Conditions for Coverage to increase donation rates and organ transplantation rates by replacing the current measures with new transparent, reliable, and objective measures". **[4]** The criteria for inclusion of an individual as a potential donor were as follows: 1) Age of the potential donor (75 years or less), death during an inpatient hospitalization, and 3) "Death that is consistent with organ donation", which includes all deaths from state death certificates with the primary cause of death listed as the ICD-10-CM codes I20-I25 (ischemic heart disease); I60-I69 (cerebrovascular disease); V-1-Y89 (external causes of death): Blunt trauma, gunshot wounds, drug overdose, suicide, drowning, and asphyxiation.

Numerator: To calculate an overall donation rate, one could employ deceased donor counts readily available on-line. We were looking to examine kidney donation rates, which entailed use of the Scientific Registry of Transplant Recipients (SRTR) standard analysis files for both donors and kidney recipients. Deceased donors were determined and then linked to recipients of at least one donated kidney to define them as a deceased kidney donor.

Calculations: Donation rates were calculated by diving the sum of all deceased kidney donors by the sum of all potential donors overall and by each stratum (age, sex, and race/ethnicity). To calculate strata specific rates, the total number of deceased donors were determined for each stratum and then divided by the total number of potential donors within the strata.

© 2024 Bragg-Gresham J et al. *JAMA Network Open.*
