# Supplementary material for: Rate of Deceased Kidney Donation From Potential In-Hospital Deaths in the US, 2003-2021
Source: JAMA Netw Open. 2024 Mar 11;7(3):e241865. doi: 10.1001/jamanetworkopen.2024.1865 (PMC10928501; doi:10.1001/jamanetworkopen.2024.1865)
Supplement: Supplement 2. — Data Sharing Statement [file jamanetwopen-e241865-s002.pdf]

Data Sharing Statement

Bragg-Gresham. Rate of Deceased Kidney Donation From Potential In-Hospital Deaths in the US, 2003-2021. *JAMA Netw Open*. Published March 11, 2024.
doi:10.1001/jamanetworkopen.2024.1865

Data
Data available: No

Additional Information
Explanation for why data not available: Readers may request these data by request from both the CDC and the SRTR websites.
